# Ultrasonography of Widespread Metastases in Advanced Gastric Signet Ring Cell Carcinoma

**DOI:** 10.3390/diagnostics15172177

**Published:** 2025-08-28

**Authors:** Xiaocong Dong, Li Zhang, Xiaohui Li, Luying Gao, Jianchu Li

**Affiliations:** 1 Department of Ultrasound, Peking Union Medical College Hospital, Chinese Academy of Medical Sciences and Peking Union Medical College, No. 1, Shuaifuyuan, Dongcheng District, Beijing 100730, China; dongxiaocong0709@163.com (X.D.); zhangli3798@163.com (L.Z.); gaoluying12@foxmail.com (L.G.); 2 Department of Ultrasound, Aerospace Center Hospital, No. 15, Yuquan Road, Haidian District, Beijing 100049, China; 3 Department of Ultrasound, Beijing Sixth Hospital, No. 36, Jiaodaokou North 2nd Alley, Dongcheng District, Beijing 100007, China; 15201242030@163.com

**Keywords:** gastric signet ring cell carcinoma, metastasis, ultrasonography, super microvascular imaging

## Abstract

Advanced Gastric Signet Ring Cell Carcinoma (SRCC) is characterized by aggressive behavior, high metastatic potential, and extremely poor prognosis. There is an urgent need for effective imaging modalities to evaluate systemic metastatic lesions and to dynamically monitor disease progression during treatment. We report a rare case of a 26-year-old female with advanced SRCC presenting with extensive systemic metastases, clinically staged as IV (cT4N3M1). High-frequency and conventional ultrasound imaging revealed metastatic lesions involving the scalp soft tissues, cervical lymph nodes, intercostal soft tissues, pancreatic-splenic hilum region, pelvic cavity, peritoneum and omentum. The ultrasonographic findings were highly consistent with contrast-enhanced computed tomography (CT) and magnetic resonance imaging (MRI) results. The patient received seven cycles of a modified BEMA regimen (oxaliplatin, leucovorin and 5-fluorouracil) combined with nivolumab. Serial ultrasound monitoring indicated continuous disease progression. Due to poor therapeutic response, the patient succumbed to acute obstructive renal failure caused by tumor progression seven months after diagnosis. This report provided a comprehensive ultrasonographic assessment of widespread and rare metastatic sites in advanced SRCC, a scenario seldom documented. The combination of high-frequency ultrasound and Super Microvascular Imaging (SMI) offered precise, radiation-free, and repeatable evaluation of both superficial and deep lesions, proving particularly valuable for real-time monitoring of treatment response in critically ill patients. These findings underscore the unique role of systemic ultrasound in enhancing metastatic detection and therapeutic evaluation for advanced SRCC.

**Figure 1:**
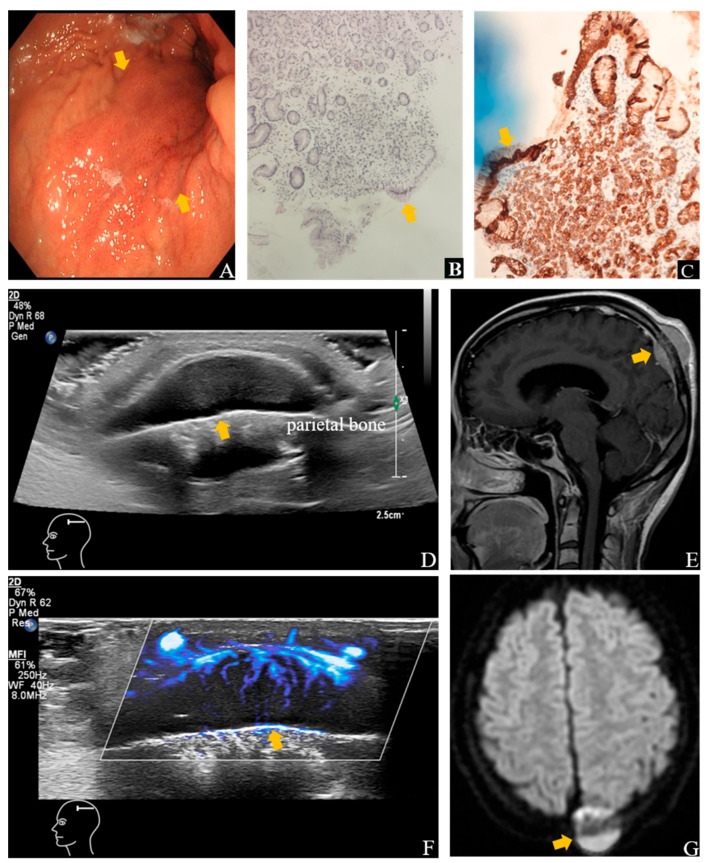
Ultrasound (US) and contrast-enhanced MRI images show the lesion in the left parietal bone. An endoscopic biopsy ((**A**), **arrow**) performed four months earlier in a 26-year-old woman with epigastric discomfort confirmed SRCC on histopathological examination ((**B**), **arrow**), Hematoxylin and Eosin staining (H&E), ×10 and (**C**), **arrow**), Immunohistochemical Staining (IHC), AE1/AE3 (+)). She complained of right intercostal pain and palpable, firm, immobile masses over the scalp, left neck, and lower abdomen. Her maternal grandmother had a history of cardia cancer. Gray-scale image showed a 3.1 × 2.9 × 0.8 cm solid mass in the subgaleal layer over the left parietal region adjacent to the skull (**arrow**), with ill-defined margins and abundant vascularity (**arrow**) on SMI (**D**,**F**). Disruption of the adjacent calvarial cortex was noted. Contrast-enhanced MRI of the head revealed a hyperintense lesion in the left parietal bone (**arrow**) with osteolytic destruction (**E**,**G**), consistent with osseous metastasis. One month later, follow-up ultrasound showed enlargement of the scalp lesion to 3.7 × 3.6 × 1.5 cm, and after two months, further progression to 5.7 × 5.1 × 2.0 cm.

**Figure 2:**
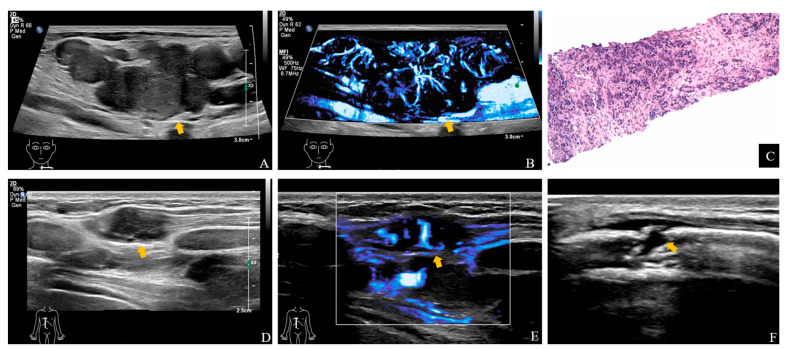
US images show metastatic lesions involving the cervical lymph nodes and intercostal soft tissues. Gray-scale image showed confluent lymph node clusters in the left cervical and supraclavicular regions (**arrow**) with increased vascular flow (**arrow**) on SMI (**A**,**B**). Fine-needle aspiration of the supraclavicular lymph node revealed infiltrating atypical epithelial cells ((**C**), H&E, ×10). Immunohistochemistry was consistent with metastatic gastric adenocarcinoma. Gray-scale images showed several solid nodules in the subcutaneous and muscular layers of the right intercostal region, the largest measuring 2.0 × 0.7 cm (**D**), **arrow**), accompanied by rich vascularity (**E**), **arrow**) and cortical disruption of adjacent ribs (**F**), **arrow**). Contrast-enhanced CT of the chest revealed bilateral pleural thickening suggestive of metastases.

**Figure 3:**
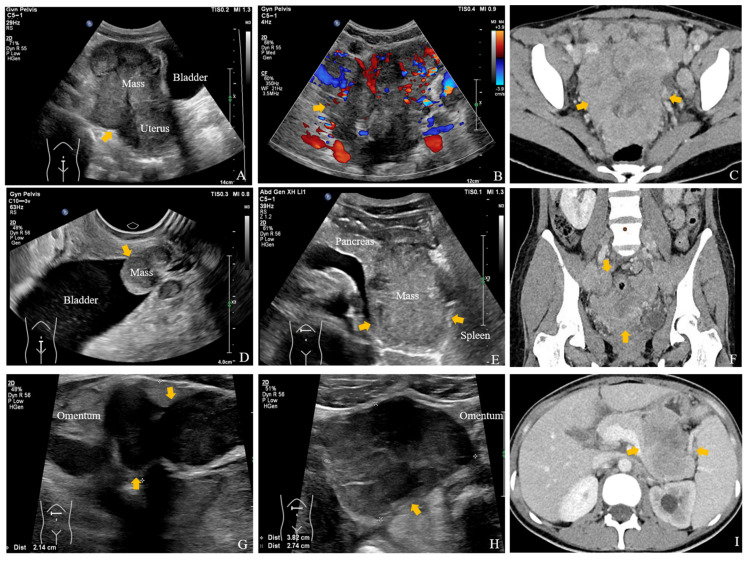
US and Contrast-enhanced CT images show metastatic lesions involving the pelvic cavity, pancreatic-splenic hilum region, peritoneum and omentum. Pelvic ultrasonography disclosed an 11.0 × 7.6 × 9.5 cm infiltrative mass involving uterus and bladder (**arrow**), with prominent vascular signals (**arrow**) (**A**,**B**). Contrast-enhanced CT showed an ill-defined heterogeneous mass in the bilateral adnexal areas (**arrow**) (**C**,**F**). Additional findings included a 1.9 × 1.6 cm vascularized nodule on the bladder wall (**D**), **arrow**), an 8.7 × 6.2 × 6.5 cm poorly demarcated mass in the pancreatic-splenic hilum region on ultrasonography (**E**), **arrow**) and contrast-enhanced CT (**I**), **arrow**), and diffuse peritoneal and omental thickening with multiple nodules (**arrow**), the largest measuring 3.8 × 2.7 cm (**arrow**) (**G**,**H**). The pelvic mass was consistent with a Krukenberg tumor, typically appearing as a heterogeneous solid lesion with potential cystic areas [1]. The irregular peritoneal thickening and omental cake appearance indicated metastatic spread [2]. The patient received seven cycles of a modified BEMA regimen combined with nivolumab. Initial treatment response was stable disease; however, follow-up showed progression at these sites, and the patient ultimately succumbed to acute obstructive renal failure due to tumor progression, which aligns with the aggressive nature and poor prognosis of advanced SRCC [3,4]. Systemic ultrasonography, combining high-frequency imaging and microvascular techniques, plays a crucial role in detecting rare metastatic sites and monitoring therapeutic response in real-time, offering an early, sensitive, radiation-free, and repeatable assessment for patients with poor general condition [5,6].
